# Analysis of the Household and Health Care System Expenditures in Bulgaria

**DOI:** 10.3389/fpubh.2021.675277

**Published:** 2021-07-02

**Authors:** Zornitsa Mitkova, Guenka Petrova

**Affiliations:** Department of Organization and Economy of Pharmacy, Faculty of Pharmacy, Medical University of Sofia, Sofia, Bulgaria

**Keywords:** public expenditures, household expenditures, healthcare expenditure, cost containment measures, Bulgaria

## Abstract

Health care systems worldwide are experiencing tremendous financial pressure because of the introduction of new targeted health technologies and medicines. This study aims to analyze and compare public and household healthcare expenditures in Bulgaria during the period 2015–2019, as well as present the major cost-containment measures implied by the government and their probable influence on the overall health care cost. Regulatory analysis of the endorsed cost-containment measures, budget analysis of public and household health care expenditures, and their extrapolations were performed. The regulatory analysis reveals that a large number of measures are introduced and valid until January 2021, considering pharmaceuticals, medical devices, and negotiations between the National Health Insurance Fund (NHIF) and Marketing authorization holders (MAHs). NHIF costs due to pharmaceuticals, food supplements, and medical devices are rising from 2015 to 2019. The overall health expenditures average per household and the average per person also grow in this period. The cost extrapolation reveals that an increase in 3-year periods is expected. Despite the implementation of variety of cost-containment measures in Bulgaria, such as HTA, ERP, discounts, and annual negotiations, The National Health Insurance Fund's (NHIF) spending on pharmaceuticals continues to rise in recent years, and further increases are expected in the next 3 years. The average expenditure per household and per person also increased, which confirms the global trend of rising medicine and outpatient services value.

## Introduction

EU countries spent about 9.6% of their GDP on healthcare in 2017. Switzerland spent the largest share (12.3%), and Turkey the lowest with 4.2%. Among EU countries, Germany spent the most on pharmaceuticals per capita (EUR 572), whereas in Ireland it is EUR 498, and in Belgium it is EUR 491. These countries spent nearly 20% more than the average EU rate. In contrast, Denmark (EUR 203), Romania (EUR 255), Estonia (EUR 262), and Poland (EUR 267) spent less on pharmaceuticals per capita (1).

Despite the implementation of a variety of cost-containment measures worldwide, pharmaceutical expenditures continue to rise. Therefore, efficient allocation of a health care budget is especially important for ensuring adequate financing of hospital services, pharmaceuticals, and physicians' care. Pharmaceuticals' expenditures capping is unlikely to affect resource allocation effectively (2). Despite some evidence from Germany, Portugal, Italy, and France, pharmaceutical expenditure capping and payback mechanisms might lead to cost savings (3).

Health care systems worldwide are experiencing tremendous financial pressure because of the introduction of new targeted health technologies and medicines. Insufficient financing might place a financial burden on households. Some studies show that the level of co-payment for health care services is exceedingly high in Bulgaria (4, 5). For medicines, it is almost 45% and creates a risk for patients' non-compliance due to financial constraints of the families (6).

This study aims to analyze and compare public and household healthcare expenditures in Bulgaria during the period 2015–2019, as well as present the major cost-containment measures implied by the government and their probable influence on the overall health care cost.

## Materials and Methods

### Design of the Study

Regulatory analysis of all cost-containment measures implemented in Bulgaria and endorsed until January 2021 in the first part of the study.

The next part of the study presents a comparative, retrospective, observational, and macroeconomic analysis of NHIF and household expenditure during the 2015–2019 periods. Both public and household expenditures are presented in terms of total and per capita. Extrapolation of expenditures is also applied to establish future tendencies.

All costs are presented in Euros based on the exchange rates of 1 Euro = 1.95 BGN in December 2021.

### Data Sources

Two databases were analyzed in this study. The National Statistical Institute (NSI) database was used to extract household health care expenditures. They were categorized into the following groups as demonstrated in Table 1.

**Table 1:** Household expenditure categories included in the analysis.

06	Health.
061	Medical products, appliances, and equipment.
061112	Pharmaceutical products.
062	Outpatient services.
062113	General practice.
062114	Specialist practice.
063	Hospital services.

The dataset of the National Health Insurance Fund (NHIF) was reviewed to extract public expenditure on pharmaceuticals, medical devices, and dietetic foods during the observed period.

### Regulatory Analysis

Cost-containment measures endorsed in the legislation until January 2021 and concerning payment of pharmaceuticals and health care services, as well as describing negotiations among main stakeholders (NHIF) and marketing authorization holders (MAHs) are systematized chronologically and explained.

### Statistical Analysis

The *t*-test *via* Excel 10 was applied to establish statistically significant differences between all household costs during the observed period.

## Results

### Cost-Containment Measures in Bulgaria

The cost-containment measures endorsed until January 2021 mainly cover NHIF expenditures for pharmaceuticals, medical devices, and dietetic food (Table 2). All measures were negotiated with MAHs and signed annually in an agreement.

**Table 2:** Cost containment measures available and actual in January 2021 in Bulgaria.

**Implemented measure**	**Brief explanation**	**Regulatory act**
NHIF financial package includes dietetic foods; medical and dental prevention and diagnostics activities; urgent medical care; rehabilitation; pregnancy; childbirth, and maternity health care; outpatient medicinal products; medical devices and dietary foods; aids, devices, and facilities for people with disabilities.	According to law NHIF pays for dietetic food when they are reimbursed in at least three of the following countries: Romania, Czech Republic, Estonia, Greece, Hungary, Lithuania, Portugal, and Spain, as well as urgent, ambulatory treatment of all citizens.	Health Insurance Law, last amended December 2020 (7)
Price control based on international price referencing (external reference pricing) with 10 reference countries.	The reference price setting was introduced for the first time in 2011 to control NHIF expenditure for all medicines on the positive drug list (PDL).	Ordinance on rules, conditions, regulations and price registration of medicinal products, last amended March 2020 (8)
Generics price capping and their link with the originator price.	Prices of generics or biosimilars should not exceed 70% of originators prices and 80% of reference biologic.	Ordinance on rules, conditions, regulations and price registration of medicinal products, last amended March 2020 (8)
Relationship between price regulation and reimbursement policies.	A positive drug list is implemented during 2007 and reimbursement is based on the lowest price per DDD (internal reference pricing) for each individual INN.	Ordinance on rules, conditions, regulations and price registration of medicinal products, last amended March 2020 (8)
Annual negotiations and price-volume agreements concerning all reimbursed medicines included in PDL.	The mechanism is applied in case of exceeding of annual amount paid by National Health Insurance Fund for medicinal products. If their expenditures exceed the planned, MAH pay-backs the exceeded amount. Cap on sales volume was implemented in 2018 to reach budget sustainability and predictability.	Ordinance on rules, conditions, regulations and price registration of medicinal products, last amended March 2020 (8)
Annual negotiations and price-volume agreements concerning all fully reimbursed medical devices for hospital care.	The mechanism is applied in case of exceeding of annual amount paid by National Health Insurance Fund for medical devices.	Health Insurance Law, last amended December 2020 (7)
HTA for new medicines before inclusion in PDL.	Positive HTA evaluation of cost-effectiveness of new medicines is obligatory.	Ordinance on rules, conditions, regulations and price registration of medicinal products, last amended March 2020 (8)
Discount of medicinal products in a group where no generics are available for treatment of chronic and oncology diseases, as well as new INNs included in PDL.	Discount should be not <10% of reimbursed price for medicinal products for 3 months period. Annual discount is applied if there is an increase in forecasted expenditures negotiated between MAH and NHIF. The discount could be 25, 50, 75, 90% depending on exceeded amount annually.	Ordinance 10 on the conditions and procedures for medicinal products payment based on Law of medicinal products in human medicines, medical devices, and dietary foods, and specific activities regulated by Health Law, last amended November 2017 (9)
Discount concerning new medicinal products before inclusion in PDL based on agreement between MAH and NHIF.	After positive HTA decision MAH and NHIF have to discuss the annual rate of discount which is obligatory.	Ordinance 10 on the conditions and procedures for medicinal products payment based on Law of medicinal products in human medicines, medical devices, and dietary foods, and specific activities regulated by Health Law, last amended November 2017 (9)

Despite the permanent endorsement of new cost-containment measures in Bulgaria, the payers' expenditure has continued to rise in recent years.

### NHIF Expenditure

We found a slight cost increase during the 2015–2019 period (Table 3) considering the reimbursement of medicinal products, food supplements, and medical devices.

**Table 3:** NHIF expenditures during 2015–2019.

**NHIF expenditure, euro**	**2015**		**2016**		**2017**		**2018**		**2019**	
	**Total annual cost**	**Cost per patient**	**Total annual cost**	**Cost per patient**	**Total annual cost**	**Cost per patient**	**Total annual cost**	**Cost per patient**	**Total annual cost**	**Cost per patient**
Pharmaceuticals	319,994,122.9	44.73	354,131,856	49.87	408,233,778	57.91	406,634,309	58.09	407,881,389	58.68
Pharmaceuticals, food supplements, medical devices	331,785,134.6	46.38	365,552,750	51.47	420,264,505	59.61	419,348,925	59.91	420,703,772	60.52
Period considering for	2015/2016		2016/2017		2017/2018		2018/2019		2015/2019	
Change in a %	10		14		−0.22		0.31		27	

**Figure 1:**
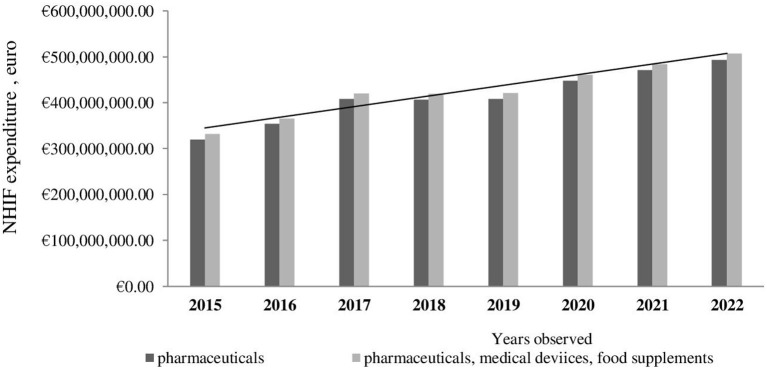
Extrapolation of NHIF expenditures to 2022.

Further cost extrapolation reveals that the NHIF cost for medicinal products as well as overall costs, including medical devices and food supplements, is expected to rise within the next 3 years (Figure 1).

### Household Expenditure

We found a slow increase in household expenditure until 2018, whereas in 2019, the increase was significant. Household expenditure in Bulgaria differs in recent years due to changes in prices and reimbursement medicines, outpatient clinical services, and hospital care. The cost could be observed from the individual person's point of view as well as the average per household (Table 4).

**Table 4:** The average household cost and cost per person.

**Average cost per household (euro)**	**2015**		**2016**		**2017**		**2018**		**2019**	
Overall health expenditures	304		315		328		358		431	
Medical products, appliances, and equipment	240		251		263		295		320	
Pharmaceutical products	217		230		241		261		281	
Outpationt services	43		39		39		39		44	
General practice	3		2		2		2		1	
Specialist care	6		5		5		5		5	
Hospital services	21		25		26		24		66	
*t*-test of all costs average per household compared within	**2015**	**2016**	**2016**	**2017**	**2017**	**2018**	**2018**	**2019**	**2015**	**2019**
*p*-value	0.1226		0.0687		0.0963		0.0598		0.0527	
Mean values	119.1	123.8	123.8	129.14	129.14	140.57	140.57	164	119.1	164
Variance	16,679	18,319	18,319	20,052	20,052	24,518	24,518	30,861	16,679	30,861
**Average cost per person (euro)**	**2015**		**2016**		**2017**		**2018**		**2019**	
Overall health expenditures	127		134		143		160		197	
Medical products, appliances, and equipment	100		107		115		132		147	
Pharmaceutical products	91		98		105		117		129	
Out-patient services	18		17		17		17		20	
General practice	1		1		1		1		1	
Specialist practice	3		2		2		2		3	
Hospital services	9		11		11		11		30	
Share of household healthcare costs from total costs	5.4		5.6		5.4		5.5		6.3	
*t*-test of all costs average per household compared within	**2015**	**2016**	**2016**	**2017**	**2017**	**2018**	**2018**	**2019**	**2015**	**2019**
*p*-value	0.0863		0.0802		0.0828		0.0457		0.0488	
Mean values	49.85	52.85	52.85	56.28	56.28	62.85	62.85	75.28	49.85	75.28
Variance	2,904.1	3,311.1	3,311.1	3,822.9	3,822.9	4,911.8	4,911.8	6,448.9	2,904.1	6,448.9

The lowest growth is found in household costs related to outpatient services, general practices, and specialist practices covered by the NHIF. The highest growth was observed for hospital services and overall costs for medicinal products, appliances, and equipment.

Despite cost variations, the *t*-test confirms that statistically significant changes occurred between 2015 and 2019 and between 2018 and 2019 with regard to the average cost per person.

**Figure 2:**
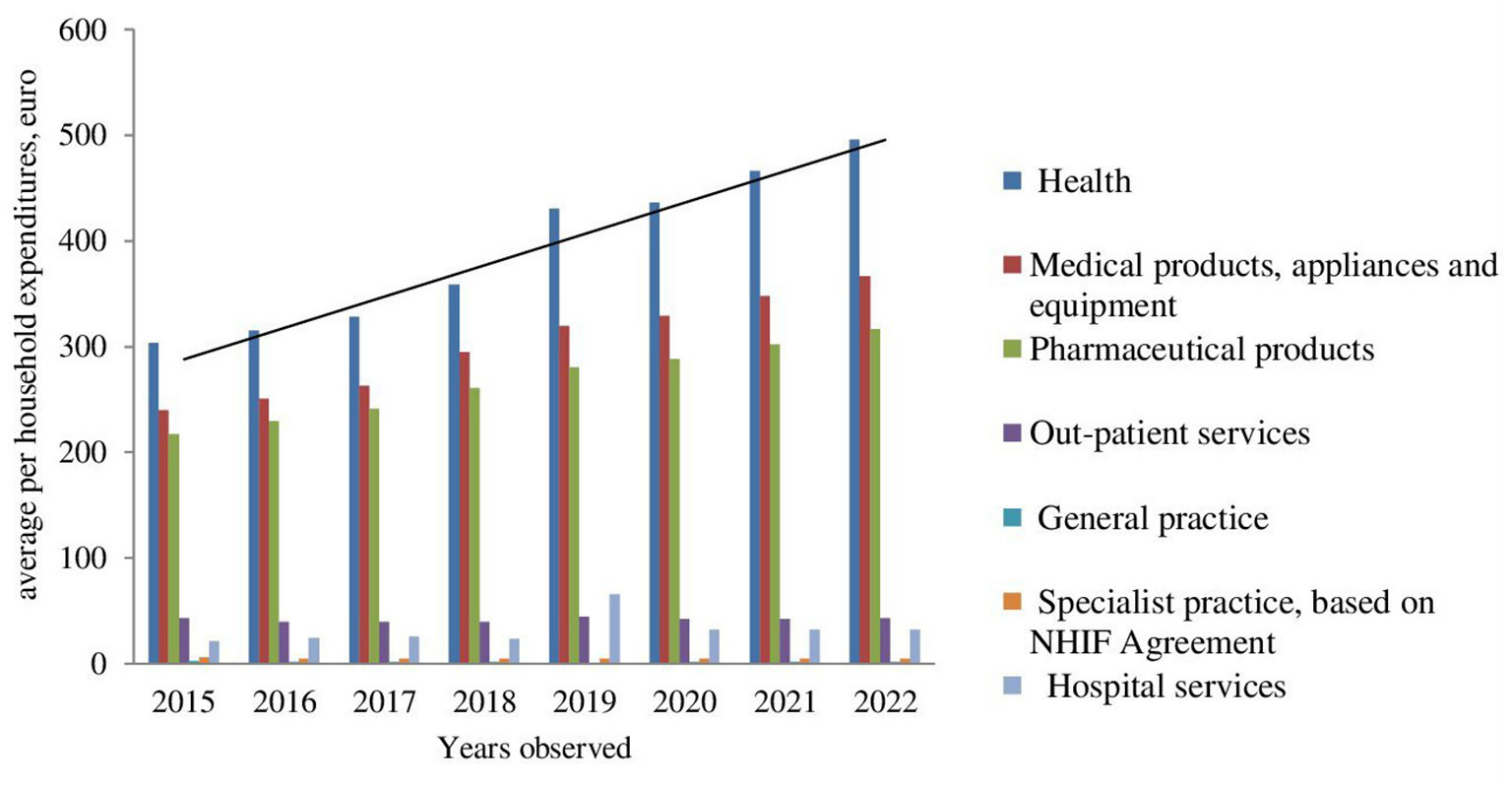
Extrapolation of average per household expenditures.

**Figure 3:**
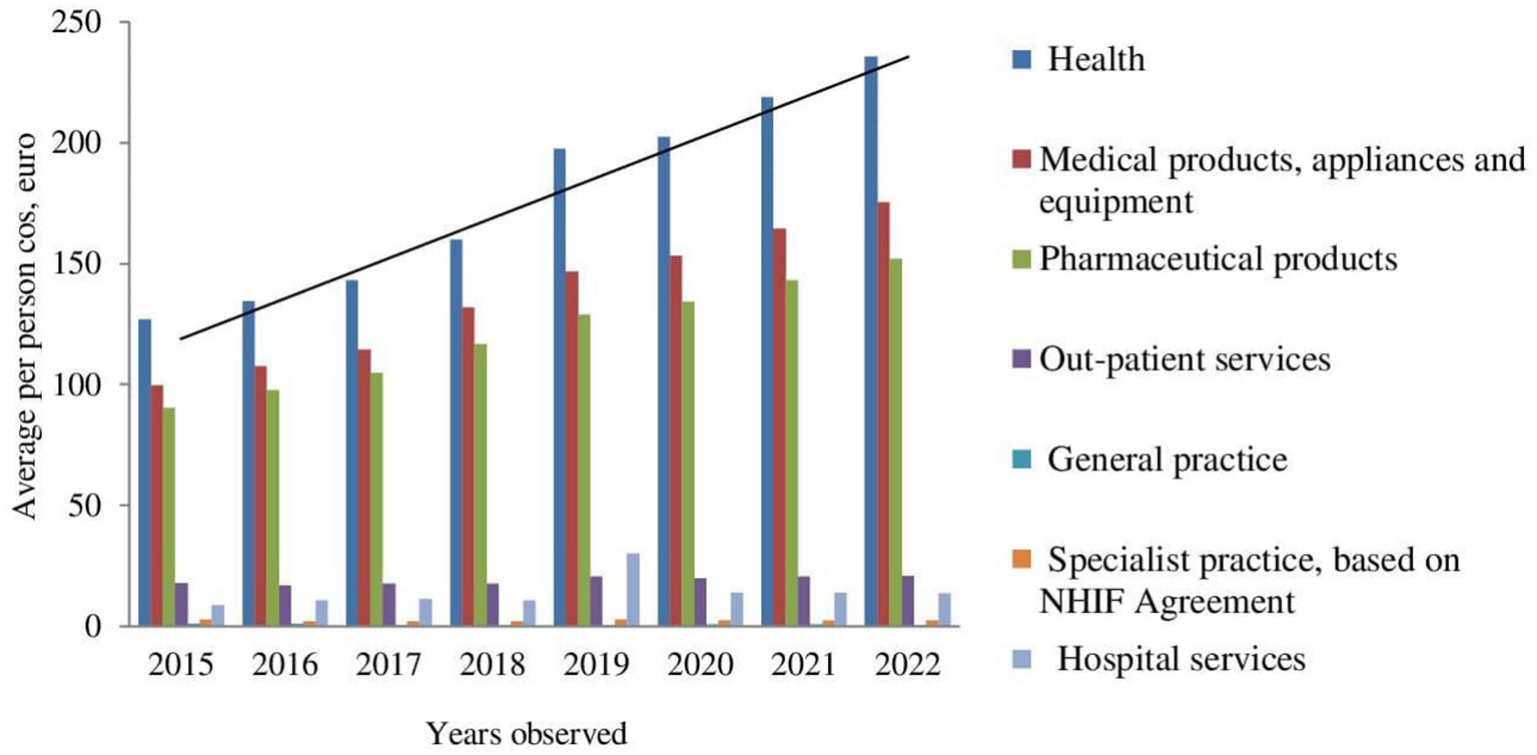
Extrapolation of average per person cost.

Further cost extrapolation reveals a rising tendency, except for the costs for outpatient services and specialist practices (Figures 2, 3).

The share of household healthcare expenditure is between 5.3 and 6.3% of all annual household expenditures.

## Discussion

Bulgaria spent EUR 1,311 per capita, or 8.1% of its GDP on health. Expenditures for pharmaceuticals are higher than the EU average and over 40% of the total health expenditure (10). Increases in income, improved household status, and affordability of medicines had a positive impact on private and public consumption growth (11).

Mediterranean countries spend less on healthcare than the EU average, both as a proportion of GDP and per capita. This is a result of lower than the EU average public funding of healthcare (12). Greece and Slovenia have the largest health care spending as percent of GDP in the period from 1995 to 2014, while the largest increase in health care expenditures was observed in Lithuania in 2014. The median value of out-of-pocket payment for health is the highest in Albania and Ukraine, and a decrease is reported in Albania, Bosnia, and Herzegovina (13).

Our results indicate that household and public expenditures in Bulgaria rose during 2015–2019 and will continue to rise in the next 3 years. The highest is the increase in hospital services, medicinal products, appliances, and equipment.

Household and public expenditures vary significantly among EU countries. The UK study reveals that healthcare costs over 9 years (2008/09–2016/17) increased significantly. Expenditures for hospital care increased by 54.1%, corresponding to increases in both activity (29.2%) and cost (15.7%), whereas community prescription grew by 45.2%, with costs falling by 24.4% due to generic utilization and HTA implementation. Overall, it accounts for over one-fifth of the total expenditure in the English NHS (14).

The Greek household health expenditure rapidly increased during the period 1988–2008 and then started to decrease. The same tendency is observed for public expenditure, household medical services, and pharmaceuticals (15).

During the period 2004–2015, the share of health expenditure as a part of the total household expenditures in Estonia, Lithuania, and Latvia remained almost stable; however, the share of health care expenditures of Polish households had increased. Compared to 2004, in 2015, Estonian, Latvian, and Lithuanian households spent less on medicinal products, appliances, and equipment, and more on outpatient services (16).

According to our results, NHIF expenditures in Bulgaria rose by 27% during 2015–2019, while household expenditures increased mainly in 2019. Within the period 2015–2019, their increase is significant, especially those for hospital treatments.

Most out-of-pocket expenditures in Albania included medicines and medical devices, followed by diagnostic and outpatient services. Hospital services and treatment abroad are less frequent but costly (17).

Different factors influence medicine prices and public expenditure worldwide. ERP is not an effective price control measure. If this methodology is implemented with minimal price revisions and a selection of basket size and countries with similar development, it could be more effective and could help ensure innovation incentives for the industry (18).

When the growth of pharmaceutical spending in France stopped, access to innovative medicines improved. The added therapeutic value of new medicines is a major reason for the reimbursement of new medicines, and institutions pay more for superior medicinal products. MAHs pay claw backs when expenditures exceed the budget, as approved by Parliament (19).

HTA and economic evaluation could lead to more rational evidence-based decision-making, possibly improving efficiency in resource allocation (20).

Despite the implementation of cost-containment measures in Bulgaria, using HTA, ERP, discount, and annual negotiations, pharmaceutical spending continued to rise recently, considering both NHIF and household points of view. Therefore, the introduction of new approaches is needed to stop and effectively control the increasing costs in a country.

The limitation of this study is the lack of data for all groups' NHIF expenditure, thus limiting the analysis only to reimbursed expenditures for pharmaceuticals, food supplements, and medical devices.

## Conclusion

Despite the implementation of a variety of cost-containment measures in Bulgaria, such as HTA, ERP, discounts, and annual negotiations, NHIF spending on pharmaceuticals has continued to rise in recent years, and further increases are expected in the next 3 years.

The average expenditure per household and per person also increased, which confirms the global trend of rising medicines and outpatient services value.

## Data Availability Statement

The raw data supporting the conclusions of this article will be made available by the authors, without undue reservation.

## Author Contributions

GP conceived and designed the investigation and wrote the discussion and conclusion. ZM collected the data and performed the data analysis and results section. All authors wrote and revised the manuscript and approved its submission for publication, confirming that the work is original.

## Conflict of Interest

The authors declare that the research was conducted in the absence of any commercial or financial relationships that could be construed as a potential conflict of interest.
